# Advances in Targeted Immunotherapy in Cancers

**DOI:** 10.3390/ijms242417475

**Published:** 2023-12-14

**Authors:** Na Luo

**Affiliations:** Department of Anatomy and Histology, School of Medicine, Nankai University, Tianjin 300071, China; luon11@nankai.edu.cn

Immunotherapy has revolutionized cancer treatment, especially the emergence of immune checkpoint inhibitors (ICIs), which has been considered as a significant breakthrough in cancer therapy. Immunotherapy utilizes a boosted natural immune system to defend and eliminate cancer cells, and various forms of immunotherapy have been developed, including oncolytic virus therapies, cancer vaccines, cytokine therapies, adoptive cell transfer, and immune ICIs [1].

Cancer vaccines employ shared or personalized tumor-associated antigens present in cancer cells to cause an immune response to recognize and react to the antigens, and destroy the cancer cells subsequently. Compared to DNA vaccines, mRNA vaccines display higher protein expression and lower toxicity level. The mRNA vaccine delivers antigen-encoding mRNA into immune cells, which induce antigen expression and the following adaptive immune responses to eliminate cancer cells. Shim et al. reviewed cancer/testis antigens (CTAs) as targets for RNA-based anticancer vaccines, which are shared tumor-associated antigens. The review summarizes the roles of CTAs in various life processes, the strategies employed for CTA-associated anti-cancer immunotherapy and clinical trials, and CTA-based mRNA vaccine for anti-cancer immunotherapy and clinical trials. Also, the authors discussed the mRNA vaccine delivery system and associated clinical trials. Although the success of other immunotherapy strategies diverted researchers’ attention from cancer vaccines, it has the advantage of targeting the intracellular antigens as well as the cell surface antigens. Moreover, it has the potential to prime new tumor-reactive immune response [2].

Chimeric antigen receptor (CAR)-T cell therapy is a dramatic innovation in cancer treatment. It collects T cells from the cancer patient and re-engineers them in the lab to produce CARs on the surface that recognize and bind to the specific antigens of cancer cells. The success of anti-CD19 CAR-T cell therapy against B cell malignancies resulted in CAR-T cell therapy entering the mainstream of cancer treatment and becoming an option for patients with blood cancer. However, with life-threatening toxicities, limited efficacy, limited persistence, and limited tumor infiltration, more strategies must be employed to limit toxicities, improve anti-tumor efficacy, and augment clinical efficacy [3]. Alsalloum et al. explored TCR-like CAR-engineered lymphocyte cytotoxicity and functional attribution against cancer/testis antigen MAGE-A4. Upon interaction with the tumor cells, the engineered lymphocytes showed increased activation markers, such as CD69, CD107a, and FasL. Moreover, T-effector genes involved in immune response and proliferation were also augmented in the engineered lymphocytes. Furthermore, the enhanced cytokine production and cytotoxicity indicated intensified anti-tumor immune response. In addition, the in vivo model against human melanoma SK-Mel-37 cells expressing MAGEA4 revealed a significant retardation of tumor growth. This study provides fundamental insights for the advancement of cancer therapy via genetically modified lymphocytes. In addition, Belovezhets et al. performed a side-by-side comparative pre-clinical analysis of CD20-specific CAR T cells encompassing different antigen-recognition modules derived from the murine antibodies, 1F5 and Leu16, and from the human antibody, 2F2. The authors conducted a comparison from the subpopulation composition and temporal dynamics, levels of activation, instant and sequential killing activity, and cytotoxicity in vivo aspects. With no statistically significant differences in subpopulation composition and temporal dynamics, levels of activation, and instant cytotoxicity, the CD20-specific CAR T cells did show distinct sequencing killing activity in vitro and cytotoxic activity in vivo. The study indicates that pre-clinical evaluation could not be based exclusively on the in vitro data; the nature of the target and the in vivo microenvironment also contribute to CD20-specific CAR T cell efficacy. Moreover, the study lays the foundation for the clinical trials utilizing these CD20-specific CAR T cells.

Immune checkpoint inhibitors (ICIs) provide a revolutionary breakthrough in the oncology field and cancer therapy. Monoclonal antibodies that target inhibitory immune checkpoints, such as CTLA-4 and PD-1/PD-L1, preventing inhibition and promoting activation and proliferation of T cells, showed promising therapeutic outcomes and have become the most widely prescribe anticancer therapies. Either ICI alone or in combination with other cancer therapies is used as first or second lines for cancer treatment [4]. Ren et al. reviewed the rationale and clinical research progress on PD-1/PD-L1-based immunotherapy alone or in combination with other modalities for the treatment of metastatic triple-negative breast cancer (mTNBC). The review evaluated the effectiveness and usefulness of immunotherapy via existing clinical trials data associated with PD-1/PD-L1 inhibitors and proposed the potential new immunotherapy strategies based on up-to-date research results.

Although patients with various malignancies benefit from ICI treatment, a low response rate still impedes its clinical application. Thus, this has given rise to the need for developing a potential approach that elevates response rates. Song et al. reviewed protein tyrosine phosphatase non-receptor 2 (PTPN2), a potential tumor immunotherapy target, in terms of its structural and functional properties, inflammatory reactions, immune-modulatory properties, and tumor immunity. PTPN2 engaged researchers’ attention when Manguso et al. identified PTPN2 as a cancer immunotherapy target using in vivo CRISPR library screening and PTPN2 augments cancer immunotherapy efficacy via IFN-γ-induced STATs phosphorylation, MHC expression, and CXCL9/10/11 secretion. The results boosted the development of specific PTPN2 inhibitor, which is under clinical evaluation for cancer therapy and paves the way for additional therapeutics to augment immunotherapy efficacy.

Accumulating evidence suggests that inflammatory status or dietary regimens influence responses to ICI therapy. However, evidence on the effects of inflammatory status or dietary regimens on ICI efficacy has been sparse [5,6]. Mahiat et al. conducted a single-center retrospective study exploring associations between systemic inflammation/nutritional status scores and outcomes in metastatic non-small-cell lung cancer (NSCLC) treated in a first-line setting with ICI in monotherapy, ICI + CT, or CT alone. The evaluation scores include Lung Immune Prognostic Index, Modified Lung Immune Prognostic Index, Scottish Inflammatory Prognostic Score, Advanced Lung Cancer Inflammation Index, EPSILoN, Prognostic Nutritional Index, Systemic Immune-Inflammation Index, Gustave Roussy Immune Score, Royal Marsden Hospital Prognostic Score, Lung Immune-oncology Prognostic Score 3, Lung Immune-oncology Prognostic Score 4, the score published by Holtzman et al., and Glasgow Prognostic Score. Although irrelevant to the treatment, the study showed that the systemic inflammation/nutritional status is associated with overall survival (OS) and progression-free survival (PFS) of metastatic NSCLC patients. Thus, the systemic inflammation/nutritional status could be a prognostic but not predictive marker in metastatic NSCLC.

The cellular and acellular components in the tumor microenvironment (TME) play significant roles in tumor initiation, growth, invasion, metastasis, and therapy responses. Neutrophils, with the well-known functions in acute phases of immune response, also release neutrophil extracellular traps (NETs) upon activation. NETs consist of chromatin DNA filaments and proteins responsible for trapping and killing extracellular pathogens [7]. There is increasing evidence showing that NETs play multiple roles in cancer development. Zhang et al. reviewed the roles of NETs in gastrointestinal (GI) tumors. The authors first described NET formation and function in the tumor microenvironment, and then summarized NET association with cancer development in gastric cancer, colorectal cancer, liver cancer, and pancreatic cancer. Furthermore, the authors discussed the methodology of NET detection and quantification. Most importantly, the authors outlined ongoing clinical trials in GI cancer patients with drugs targeting NET release. The study facilitated the understanding of the mechanisms of NETs acting on tumors, and NETs may be an emerging research hotspot for GI cancer treatment.

Immunotherapy has created a remarkable breakthrough for cancer therapy. However, the low response rate, limited clinical efficacy, severe systemic toxicities, etc., have restrained its application. Therefore, exploring the prospective approaches to circumventing the drawbacks is urgent to boost the profound progression of immunotherapy.
